# Improvement in Storage Stability and Physicochemical Properties of Whole-Grain Highland Barley Pulp Prepared by a Novel Industry-Scale Microfluidizer System in Comparison with Colloid Milling

**DOI:** 10.3390/foods13152316

**Published:** 2024-07-23

**Authors:** Hong Zhu, Wenjie Xu, Tianyu Zhang, Tao Jin, Bing Fang, Ju Qiu

**Affiliations:** 1 Key Laboratory of Precision Nutrition and Food Quality, Department of Nutrition and Health, China Agricultural University, No.17 Tsinghua East Road, Haidian District, Beijing 100083, Chinabingfang@cau.edu.cn (B.F.); 2 Institute of Food and Nutrition Development, Ministry of Agriculture and Rural Affairs, Beijing 100081, China; zhuhong@caas.cn; 3 Food Science College, Tibet Agriculture & Animal Husbandry University, Nyingchi 860000, China; 4 Tibet Academy of Agriculture and Animal Husbandry Sciences, Lhasa 850000, China

**Keywords:** whole-grain highland barley pulp, microfluidizer, colloid mill, stability, β-glucan

## Abstract

The aim of this study was to assess the advantages of an industry-scale microfluidizer system (ISMS) to prepare whole-grain highland barley pulp (WHBP) compared with colloid milling. Storage stability was evaluated by particle size, gravity separation stability, and rheological properties, as well as the microstructure observation by scanning electron microscopy (SEM) and confocal laser scanning microscopy (CLMS). The results showed that colloid milling failed to effectively homogenize the material, while ISMS sample surfaces were compact and smooth at higher pressures according to visual observation and SEM. The Turbiscan stability index of WHBP by ISMS was much lower as a result of colloid milling, demonstrating ISMS can improve WHBP stability. WHBP by colloid milling displayed a three-peak particle size distribution pattern, while a single-peak pattern was evident after ISMS treatment. A higher shear rate decreased the apparent viscosity, suggesting that WHBP was a shear-thinning fluid. According to CLMS, ISMS can successfully improve homogenization by disrupting the structures of oil bodies, proteins, and starches. The WHBP prepared by ISMS exhibited a higher β-glucan level than that prepared by colloid milling, and showed a significant increase in β-glucan level with ISMS pressure. These findings indicate that using ISMS to produce WHBP is viable for enhancing its storage stability and nutritional value.

## 1. Introduction

The cultivation area of highland barley (HB, *Hordeum vulgare* var. *coeleste Linnaeus*) on the Qinghai Tibet plateau covers approximately 0.27 million hectares. Since the unique nutritional value of HB is superior to that of normal cereals, it is attracting increasing attention due to its significant economic promise. According to Wang et al. (2011), HB exhibits higher protein and unsaturated fatty acids content compared to several other cereal crops [1], while Zhang et al. (2019) reported that HB was abundant in various nutritional components, including polyphenols, arabinoxylan, and β-glucan [2]. These constituents are attributed to the antibacterial, anti-tumorigenic, and antioxidant properties of HB [3,4]. Furthermore, the estimated glycemic index (GI) of HB starch ranges from 39.4 to 47.5, which is below the threshold of 55. Consequently, HB can be classified as a low-GI food option for people with diabetes [5]. Incorporating whole-grain HB into the diet can significantly enhance the populations of beneficial intestinal bacteria, including *Fusicatenibacter*, *Desulfovibrio*, and *Bifidobacterium*, which can generate short-chain fatty acids, such as butyrate [6]. Thus, the complete utilization of whole-grain highland barley pulp (WHBP) holds promise as a viable HB milk alternative. This approach allows for the full utilization of HB without the need for filtration, resulting in optimal raw material use while decreasing environmental contamination. However, minimal studies are available involving the use of WHBP. The primary obstacle during WHBP preparation is inadequate pulverizing equipment. Using conventional pulverizing equipment to process WHBP typically yields a coarse flavor and the result lacks stability due to insufficient fine grinding of the materials. This can deplete the essential nutrients in the HB and increase processing costs and energy usage.

The potential use of microfluidization in cereal-based products, namely cereal bran suspension, dairy products such as milk and cheese, and beverages like fruit juice, has been investigated [7,8]. This method offers several benefits, such as continuous operation, minimal nutrient loss, short processing times, low operating temperatures, and the absence of external chemicals, showing significant application potential for fiber-rich food production. It also enhances product performance, for both suspension and emulsion systems, by reducing coagulation time, retrogradation rate, and syneresis, and non-thermally inactivating microbes and enzymes [9]. For example, microfluidization treatment improves the viscosity and stability of wheat bran suspension, which are important for the application of bran in food systems [10]. However, few studies have focused on the microfluidization of suspensions of complex real food matrices, such as whole-grain HB, which consists of starch (49.14~68.62%), protein (6.35~23.40%), and dietary fiber (18.16~21.46%). Regarding WHBP, microfluidization also offers a promising method for the formation of complexes between starches and β-glucan, and has the potential to create a new functional resistant starch ingredient with increased viscosity and improved water-holding properties.

The existing microfluidization technology is primarily utilized in laboratory settings, with limitations imposed by its low output (<36 L/h for the Microfluidizer^®^ series) and narrow flow channels (<200 μm for the Microfluidizer^®^ series) [11]. The devices encounter difficulties in effectively processing certain materials characterized by large particle sizes, such as dietary fiber. A recently developed industry-scale microfluidizer system (ISMS) consists of a pre-pulverizer and an industry-scale microfluidizer. The ISMS consolidates the crushing, grinding, and dispersion stages of conventional processing techniques into a unified process. This novel technology effectively addresses the various existing microfluidization constraints. Consequently, it is well-suited for particle size reduction of cereal-based or fiber-rich products during industrial processing. The ISMS has been innovatively used to produce a spontaneously stable whole soy milk and whole peanut milk without filtering residues, improving the physical stability with increasing pressure [11,12]. Furthermore, whole sweet corn slurry has been also prepared using ISMS, which improved the resistance of the whole corn slurry to gravitational separation [13]. The spontaneous stability of all the whole-grain products mentioned above was related to the decreased particle sizes of macromolecules, such as dietary fiber. The ISMS may also be used to pulverize other frequently consumed food and beverages. Given the distinct composition of HB compared to soybeans, sweet corn, and peanut, it is worthwhile to investigate the viability of using ISMS to produce WHBP.

This study aimed to determine the efficacy of ISMS in transforming HB into a whole-grain beverage that exhibits excellent sensory characteristics and stability. Given colloid milling is a promising technology for plant-based beverage preparation and dietary fiber modification, colloid milling was chosen as a comparison for ISMS in this study. Storage stability was evaluated by visual observation for 30 d under room temperature. Particle size analysis, scanning electron microscopy, and confocal laser scanning microscopy were employed to investigate the microstructure of WHBP, and gravity separation stability was analyzed by static multiple light scattering. Rheological parameters were obtained from the flow curves, derived using the power law model. In addition, the β-glucan content, as an important factor for both stability and nutritional value, was tested. Consequently, this research may provide new technology for producing superior WHBP, enhancing the sustainability and nutritional value.

## 2. Methods and Materials

### 2.1. Materials and Reagents

The HB was provided by Tibet Dechen Sunshine Manor Ltd. (Shigatse, Tibet, China), while Sigma-Aldrich Chemical Ltd. (Shanghai, China) supplied the Nile Red, Rhodamine B, and FITC. The dimethyl sulfoxide (DMSO) was obtained from Rhawn Ltd. in Shanghai, China, while Aladdin Ltd. (Shanghai, China) provided the ProClin^TM^ 300 and Megazyme Ltd. (Bray, Ireland) supplied the β-glucan Assay Kit (Mixed Linkage). The PBS solution was purchased from Saint-Bio Ltd. (Shanghai, China).

### 2.2. WHBP Preparation

#### 2.2.1. WHBP Preparation via the ISMS

An ISMS (FJ-3037D, Beijing Collaborative Innovation Food Technology Co., Ltd., Beijing, China) was employed to treat HB and water (5% and 10% (*w*/*w*) mass ratio) once at 30 MPa, 60 MPa, and 120 MPa, separately. This ISMS consists of a pre-pulverizer and an industry-scale microfluidizer. The pre-pulverizer, which uses wet milling, consists of a stator–rotor structure for crushing milling. The samples were heated at 90 °C for 20 min to obtain the WHBP specimens (WHBP_M-5-30_, WHBP_M-5-60_, WHBP_M-5-120_, WHBP_M-10-30_, WHBP_M-10-60_, and WHBP_M-10-120_). Next, several samples were freeze-dried to obtain a powder, while the remaining specimens were stored at 4 °C. Then, a preservative of 0.05% ProClin^TM^ 300 (*w*/*w*) was introduced to restrict the growth of microorganisms.

#### 2.2.2. WHBP Preparation Using Colloid Mill

Before colloid milling, two dry-milling devices were employed for pre-treatment. An airflow pulverization instrument (QLM-80K) from Shangyu City Heli Powder Engineering Co., Ltd. (Zhejiang, China) and a high-speed grinder supplied by Yongkang Zhaoshen Electric Co., Ltd. (Shanxi, China) were used to grind the HB samples, which were passed through an 80-mesh or 300-mesh sieve, respectively. Then, 500 g of each sample was collected. A colloid mill (MK module, IKA, Staufen, Germany) was used to grind the HB and water mixture (total ratio of 5% (*w*/*w*)) to acquire the WHBP. The treated WHBP was heated at 90 °C for 20 min in a pot to obtain the WHBP_CM-80_ and WHBP_CM-300_ samples. Similar to the procedure mentioned in Section 2.2.1, part of samples was freeze-dried, and the remainder was stored at 4 °C with a preservative of 0.05% ProClin^TM^ 300 (*w*/*w*).

### 2.3. Storage Stability

Storage stability was evaluated according to Li et al. [14], with some modification and improvement. The WHBP samples were incubated for 30 d at 25 °C, while their appearance was monitored at 1 d, 3 d, 5 d, 10 d, 20 d, and 30 d.

### 2.4. Scanning Electron Microscopy (SEM)

To examine the WHBP microstructure, the samples were freeze-dried, attached to a metal sample holder with double-sided cellophane tape, and gold-sprayed using an E-1010 ion sputter (Hitachi, Tokyo, Japan). SEM (SU8020, Hitachi Co., Ltd., Tokyo, Japan) was used for microstructural observation at 400× and 3000× magnification according to a method reported by Lu et al. [15].

### 2.5. Static Multiple Light Scattering (S-MLS)

The gravity separation stability of the WHBP was monitored at 25 °C using a Turbiscan Tower supplied by the Formulation Company (Toulouse, France). It used S-MLS for physical stability assessment during storage for 12 h at a scanning interval of 100 s. The function spectra depicting the relationship between the scanning height and near-infrared backscatter (BS) intensity were obtained after sample scanning. The flocculation and emulsion agglomeration stability during storage were assessed according to the ΔBS, while the Turbiscan easy software (version 3.1) was used to calculate the Turbiscan stability index (TSI) as described by Qi et al. [16].

### 2.6. Particle Properties

A laser diffraction particle size analyzer (Mastersizer 3000) provided by Malvern Instruments Co., Ltd. (Worcestershire, UK) was used to determine the WHBP distribution and particle sizes during storage. The specific parameters were set based on a previous study with some modification [17]. The parameters included a 1.590 particle refraction index, a 0.001 particle absorption rate, and deionized water as a dispersant at a 1.330 refractive index.

### 2.7. Rheological Characteristics

A rheometer supplied by TA Instruments (New Castle, DE, USA) with a parallel geometric plate (60 mm in diameter) was used to ascertain the WHBP rheological characteristics according to a method by Li et al. [11]. Next, 16 mL of the WHBP was placed on the plate, after which the rheological properties were assessed at 25 °C and a shear rate ranging between 0.1 s^−1^ and 100 s^−1^. The power law model (Equation (1)) was used to fit the flow curves obtained during the experiment:

(1) $$\sigma =k\cdot \gamma^{n}$$

where *σ* represents the shear stress (Pa), *k* denotes the consistency coefficient (Pa•s^n^), *γ* signifies the shear rate (s^−1^), and *n* represents the flow behavior index.

### 2.8. Confocal Laser Scanning Microscopy (CLSM)

CLSM (Model Zeiss LSM900, Carl Zeiss, Jena, Germany) was used to examine the spatial distribution of the oil droplets, protein particles, and starch in the WHBP based on a method described in previous research [18]. The starch, protein, and oil were stained with 0.1% FITC (dissolved in DMSO), 0.1% Rhodamine B (dissolved in DMSO), and 0.1% Nile Red (dissolved in DMSO), respectively. The samples and dye were mixed at a volume ratio of 10:1, added to a culture dish, and assessed at a 40× oil immersion objective. The CLSM images were obtained at excitation wavelengths of 488 nm, 546 nm, and 530 nm and suitable emission channels. The Zeiss ZEN 3.1 (Blue Edition) imaging software (Carl Zeiss, Germany) was used to acquire the confocal micrographs.

### 2.9. Determination of the β-Glucan Content

The content of β-glucan was quantified using a mixed linkage β-glucan kit (Megazyme, Bray, Ireland) based on a method reported by Prins et al. [19]. After incubation for 5 min in a boiling water bath, samples were mixed by vibration. All other steps were as described in the instruction manual provided in the kit.

### 2.10. Statistical Analysis

Each experiment was repeated three times. One-way analysis of variance (ANOVA) and the SPSS software (version 16.0, IBM Corporation, Chicago, IL, USA) were employed for statistical analysis. Results were assessed using the least significance difference (LSD) test (*p* < 0.05) and the mean ± standard deviation are presented.

## 3. Results and Discussions

### 3.1. Visual Characteristics and Morphology

**Figure 1:**
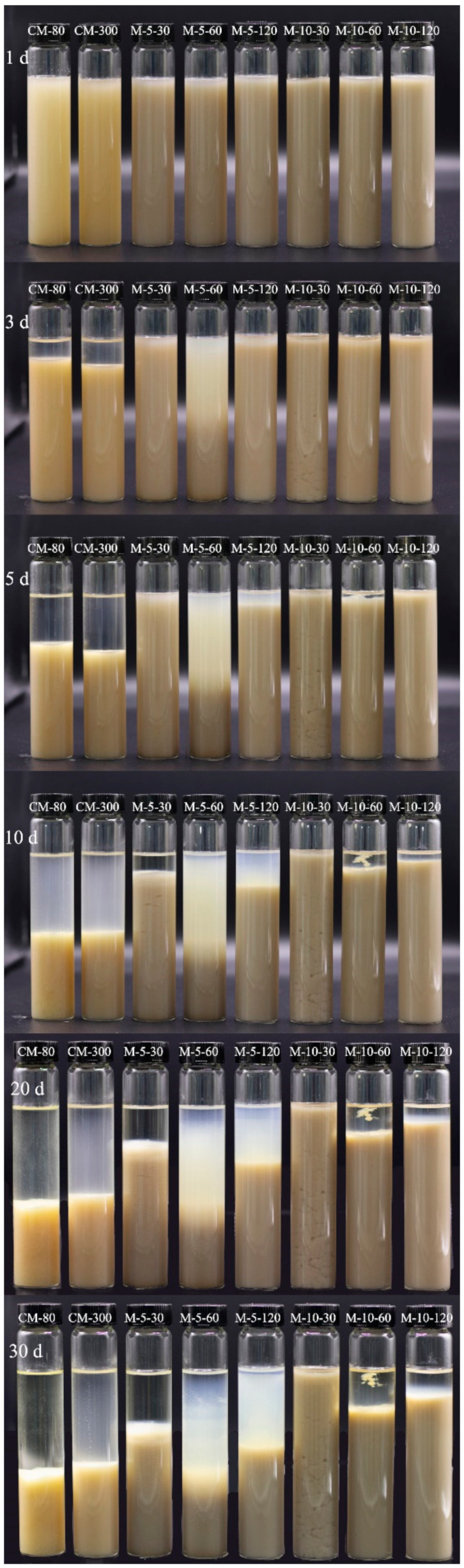
The stability of the WHBP samples prepared via colloid milling and ISMS during storage for 30 d at 25 °C.

Creaming and sedimentation pose significant challenges to the stability of cereal-based beverages. This is primarily due to the precipitation of coarse fibers and other macromolecules, as well as the likelihood of lipids floating on the surface. Consequently, stabilizers and filtration are commonly employed during cereal-based beverage manufacturing to avoid sedimentation, while emulsifiers are incorporated to restrict lipid floating. The WHBP prepared at 30 MPa, 60 MPa, and 120 MPa using ISMS spontaneously remained stable for at least 3 d at 25 °C without requiring an emulsifier or stabilizer, while the samples obtained via colloid milling were distinctly stratified from 3 d (Figure 1). At 10 d, all the samples were unstable, while the WHBP obtained via colloid milling was almost completely precipitated. Although the WHBP samples treated at 60 MPa and 120 MPa using ISMS exhibited sedimentation, no stratification was evident. This may be because some small unprecipitated material particles were dispersed in the upper pulp layer. Samples treated at 30 MPa by ISMS showed gelation.

**Figure 2:**
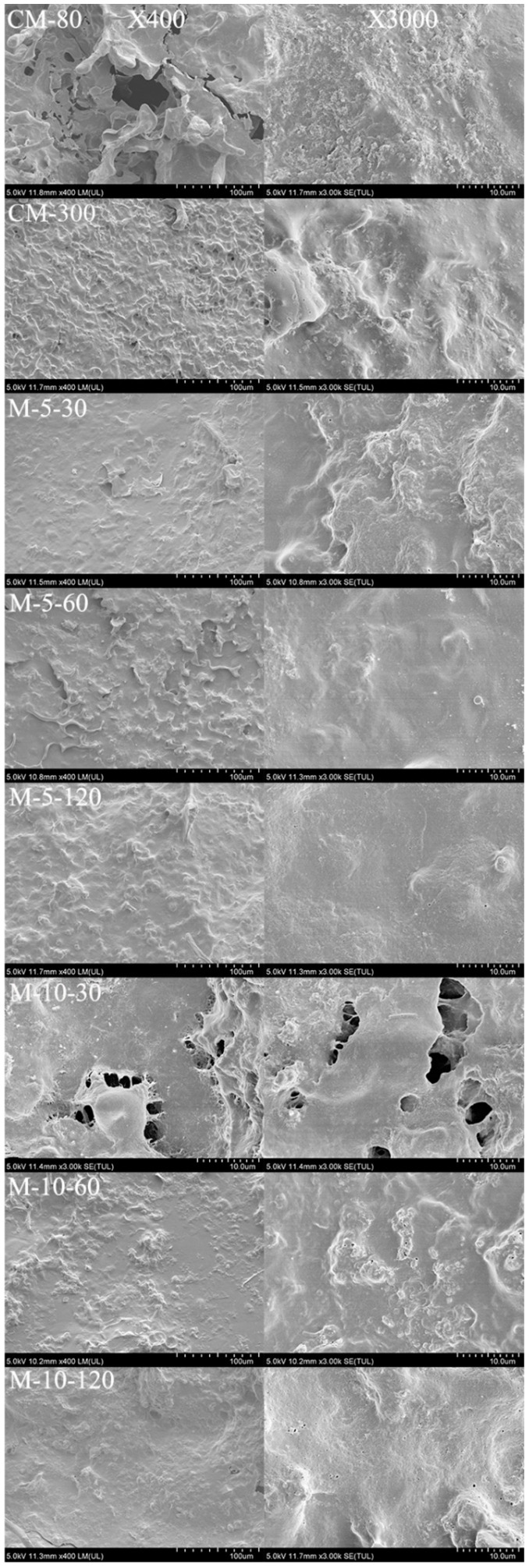
The SEM images of the WHBP samples obtained via colloid milling and ISMS.

SEM observation further clarified the effect of ISMS and colloid milling treatment on the structural particle arrangement in the WHBP samples (Figure 2). The microstructures of the samples were examined at 400× and 3000× magnification. WHBP_CM-80_ and WHBP_CM-300_ displayed irregularities, showing the distinct presence of particles (Figure 2). Although WHBP_CM-300_ exhibited a smaller average particle size, larger particles were observed at 3000× magnification, indicating that colloid milling failed to effectively homogenize the material. The ISMS sample surfaces were compact and smooth at higher pressures, while larger particles disappeared gradually. A higher operating pressure reduced the sizes of these structures due to more significant plant tissue disruption during microfluidization, which enhanced the stability [13].

### 3.2. Gravity Separation Stability

**Figure 3:**
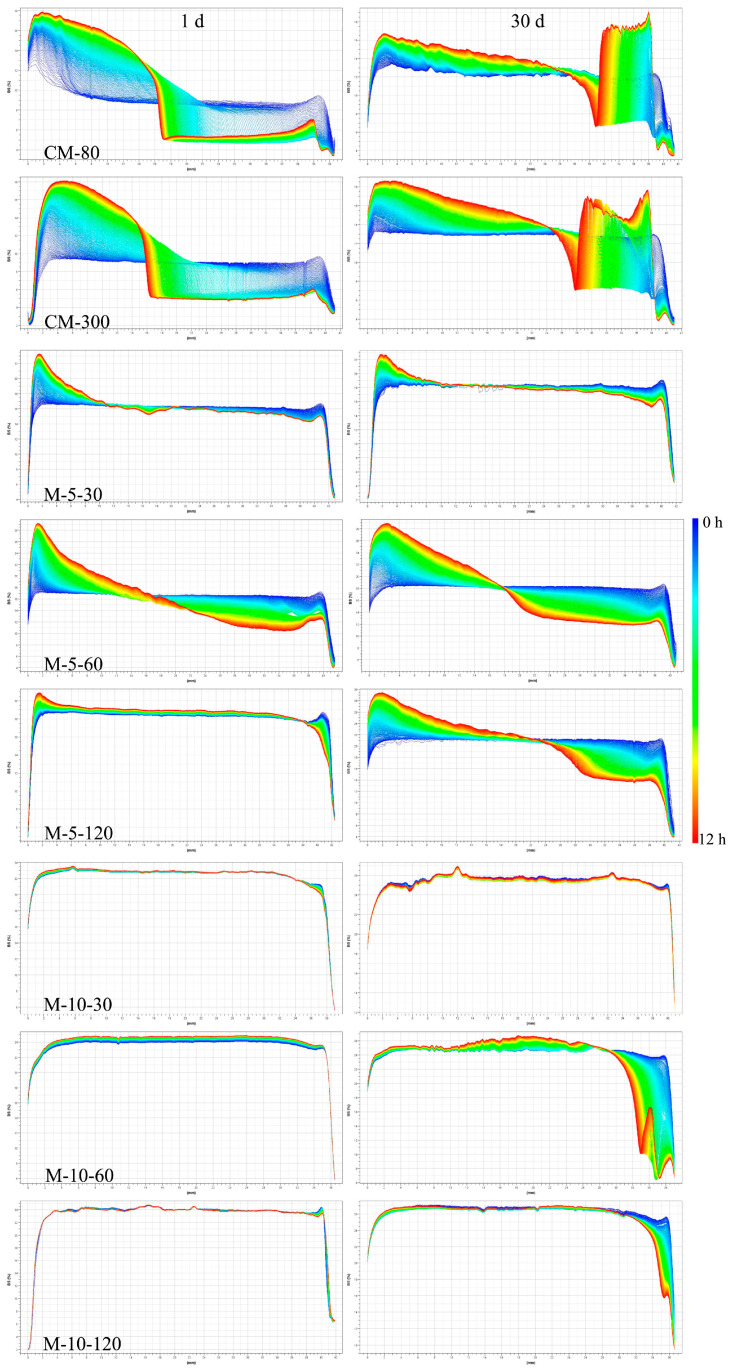
The effect of colloid milling and ISMS exposure on the physical WHBP stability.

To accurately demonstrate the storage stability variation, BS light intensity measurements were required due to the WHBP’s opacity. Lower and higher particle densities are partially represented by negative or positive peaks (Figure 3), which were attributed to emulsion degradation, creaming, or gravitational separation. The x-axis denotes the bottle height, while a more significant amplitude of the line deviating from the baseline signifies low sample stability.

The non-overlapping curves reflect the instability of the WHBP prepared via colloid milling (Figure 3). Layering and phase separation induced increased BS (ΔBS) intensity on the left, decreasing the upper layer liquid concentration. The intensity variation on the right represents the sedimentation of the unstable particles [20]. At 1 d and 30 d, significant fluctuations were evident at both ends of the spectrum of the WHBP samples prepared by colloid milling, showing irregular distribution of the particle sizes. At 1 d, the curves of the WHBP obtained via ISMS began to overlap at a higher pressure or higher concentration. All the curves exhibited overlapping at 120 MPa, demonstrating good stability.

**Figure 4:**
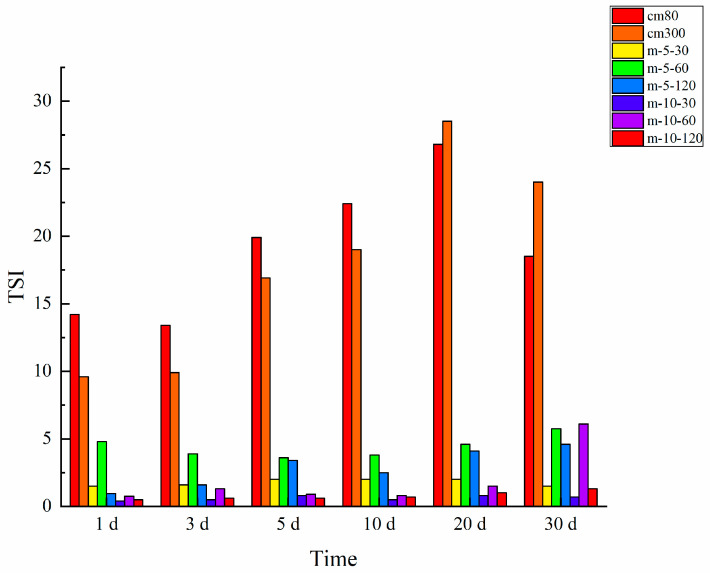
TSI values of the WHBP samples obtained via colloid milling and ISMS.

The TSI reflects the destabilization processes in a mixture, with a higher value reflecting substandard stability [21]. Compared to colloid milling, the TSI values of WHBP prepared by ISMS were much lower, demonstrating ISMS could improve the stability of WHBP (Figure 4). Among the ISMS-treated samples, the TSI declined at a higher pressure, indicating enhanced stability. Prolonged storage caused a gradual rise in the TSI values, corresponding to a decline in the emulsion stability.

### 3.3. Particle Characteristics

**Figure 5:**
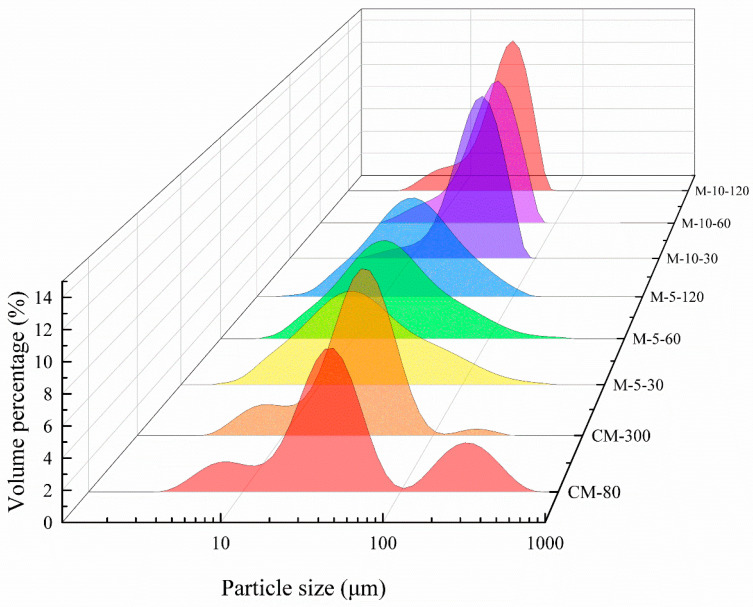
The particle size distribution of the WHBP prepared via colloid milling and ISMS.

The stability of liquid substances such as beverages and emulsions are crucially determined by the specific particle size dissemination. Figure 5 illustrates the particle size distribution curve. WHBP_CM-80_ and WHBP_CM-300_ displayed a three-peak particle size distribution pattern, with peaks at approximately 3.9 μm, 28.3 μm, and 355.7 μm, while single-peak patterns were evident after ISMS treatment. The group treated with ISMS showed a prominent peak at about 10 μm. This demonstrated that the ISMS substantially affects the pulverization of both large and small particles. A previous study found that oil-body monomers presented particles ranging from 0.1 μm to 1 μm [22], while the middle particle size peak from 1 μm to 10 μm denoted oil-body coalescence or protein aggregates [23]. The peak corresponding to the largest particle size, which varied between 50 μm and 200 μm, primarily comprised particle aggregates [24] or fibers [25,26]. This finding revealed that the macromolecules in WHBP, such as starch and dietary fiber, were broken under the effect of ISMS, leading to a small particle size and single-peak pattern. As the ISMS pressure increased, the disruption of particles intensified, changing the particle size and pattern [26].

Table 1 presents the average particle size of WHBP during storge. WHBP_CM-80_ displayed the largest particle sizes of all the samples, possibly due to significant particle and cellular tissue agglomeration. The average particle size of the WHBP obtained via conventional grinding was the highest of all the samples, particularly from 20 d to 30 d, which could be attributed to macromolecular substance agglomeration. A gradual increase was evident in the average particle sizes of samples prepared by colloid milling as the storage duration was extended. Among ISMS-treated samples, the average particle size was basically unchanged during storage. The higher the pressure of ISMS, the smaller the average particle size. At 120 MPa, the average particle size reached around 20 μm. This may be because ISMS destroys the molecular structure of starch, fibers, and proteins, making aggregation difficult, which helps to maintain the stability of WHBP.

### 3.4. Rheological Properties

**Figure 6:**
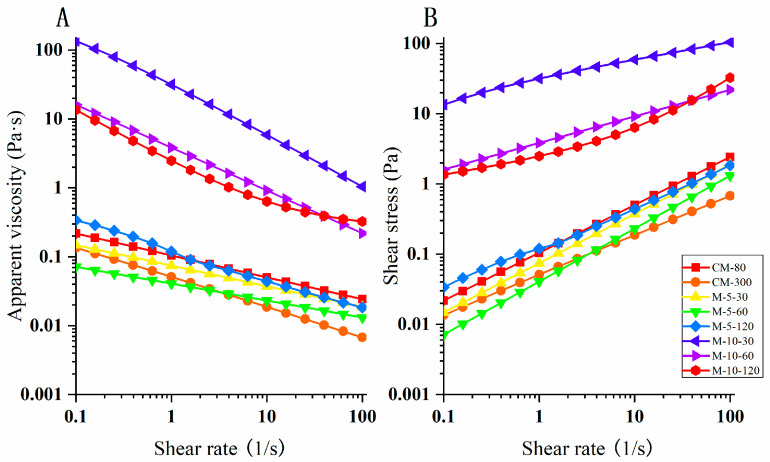
The rheological characteristics of the WHBP prepared via colloid milling and ISMS. The shear rate impact on (**A**) the apparent viscosity curve and (**B**) the shear stress.

The mouthfeel, texture, and processing of food and beverages are influenced by their rheological characteristics. Figure 6 shows the rheological attributes of the WHBP in this study, while Table 2 lists the rheological parameters obtained from the flow curves, which include the flow behavior index (*n*) and consistency coefficient (*K*). These criteria were derived using the power law model (R^2^ > 0.99) to fit the experimental data. A higher shear rate decreased the apparent viscosity values of the WHBP samples (Figure 6A), indicating shear-thinning (*n* values < 1), suggesting that the specimens were non-Newtonian fluids. Furthermore, a higher shear rate increased the sample shear stress (Figure 6B), showing that all the samples exhibited pseudoplastic fluid properties after shear-thinning. A *n* value demonstrated more significant shear-thinning [27], which increased at a higher ISMS operating pressure, as reflected by an *n*-value decline. K can be considered a criterion of viscosity. As can be seen in Table 2, the K values decreased with increasing pressure after ISMS, except for WHBP_M-5-120_. Our finding was similar to a previous study of the complex system of starch and dietary fiber [28]. This phenomenon could be due to the alteration in the macromolecular organization of the mixture system under variable shear rates and applied pressures. This was also consistent with the change in particle size. Some researchers suggested that the starch granules disintegrated, their structure was loosened, and the entanglement point between molecules decreased under higher pressure, leading to a decrease in viscosity [28,29].

### 3.5. CLSM

**Figure 7:**
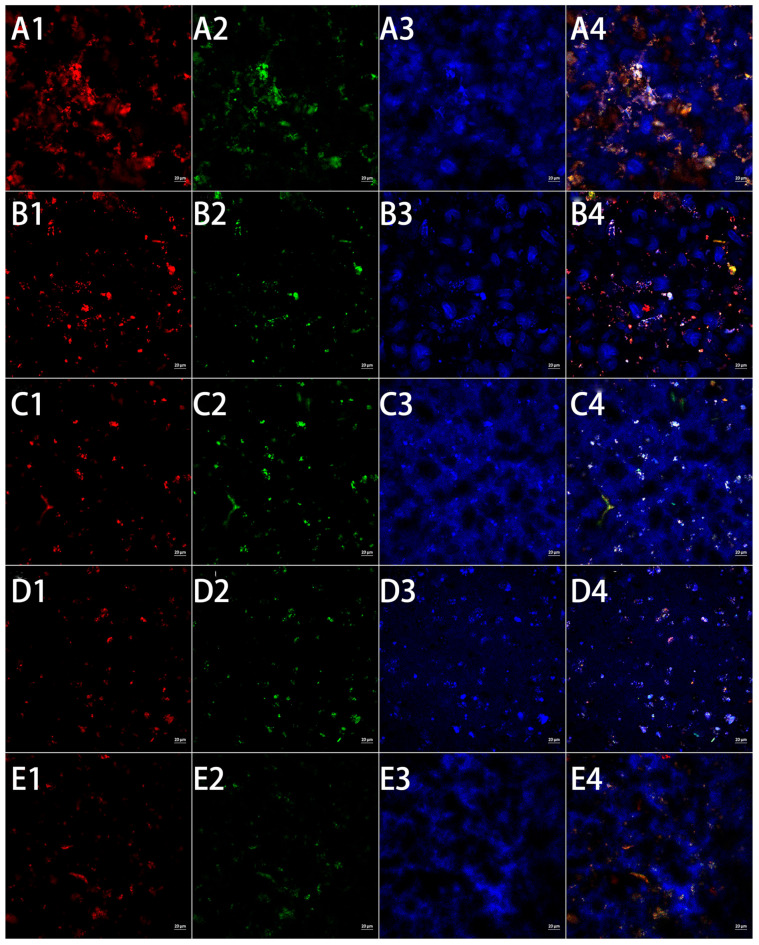
(**A**,**B**) The CLSM visualization of the WHBP prepared via colloid milling and ISMS treated at pressures of (**C**) 30 MPa, (**D**) 60 MPa, and (**E**) 120 MPa. Numbers 1, 2, 3, and 4 represent oil staining, protein staining, starch staining, and the combined image of 1, 2, and 3, respectively.

CLSM was usually used to understand the interaction of components, and especially the interaction between protein and oil. Therefore, CLSM was also used to further explore the potential physical stability mechanism of WHBP. The CLSM images showed distinct differences between the particle distribution and morphology of the WHBP samples and those immediately after treatment. Figure 7(A1–E1,A2–E2,A3–E3,A4–E4) show the oil droplet distribution (stained red), protein particles (stained green), starch (stained blue), and a combined image (overlapping areas in yellow), respectively. Substantial partial protein and oil droplet agglomeration were evident in WHBP_CM-80_ and WHBP_CM-300_ (Figure 7(A1–E1,A2–E2)). Although WHBP_M-5-30_, WHBP_M-5-60_, and particularly WHBP_M-5-120_ exhibited adequate small-particle distribution, their sizes declined at higher operating pressures, indicating increased particle disruption. Therefore, ISMS successfully reduced fat droplet and protein particle sizes [11,13]. Starch granules were evident in both WHBP_CM-80_ and WHBP_CM-300_ (Figure 7(A3–E3)), demonstrating that colloid milling failed to destroy the starch structure, even with the finer particles. Contrarily, no visible starch granules were observed in the ISMS-treated samples, indicating that the granular structure was destroyed. The pores gradually became smaller as the pressure increased, suggesting that the ISMS prevented the conversion of starch into a gel. Previous research showed that ISMS disrupted the starch granules in potato starch while decreasing their degree of crystallinity [30]. Overall, ISMS can successfully improve homogenization by disrupting the structures of oil bodies, proteins, and starches, which increases the solubility and stability of WHBP.

### 3.6. β-Glucan Content

**Figure 8:**
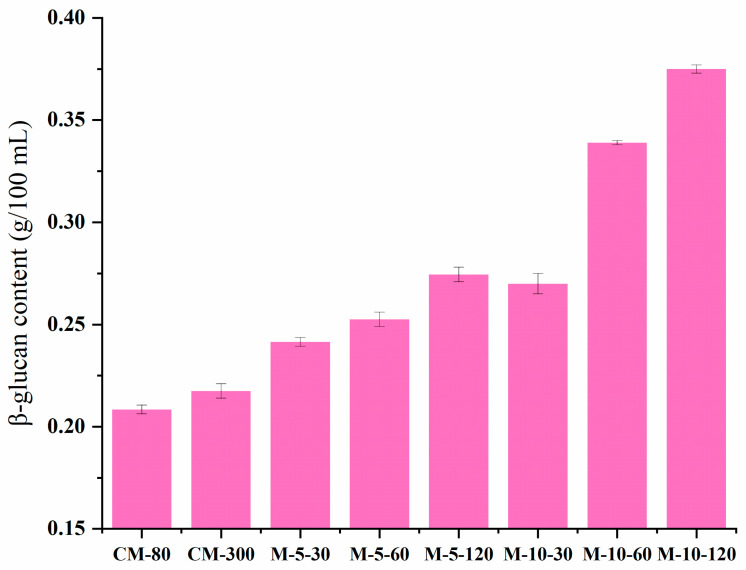
The β-glucan content in the WHBP samples obtained via colloid milling and ISMS.

The β-glucan content in the WHBP samples was 0.21–0.22 g/100 mL when using colloid milling and 0.24–0.37 g/100 mL after ISMS treatment, with significant differences between the two groups (Figure 8). The β-glucan content increased significantly at a higher ISMS treatment pressure. Tu et al. (2014) showed that microfluidization caused dietary fiber redistribution from an insoluble to a soluble fraction [31]. It was reported that the microfluidizer treatment can not only reduce the particle size of fiber, but also change the structure of the fiber. Another similar study revealed that microfluidization reduced the fiber particle size while modifying its structure [32,33].

## 4. Conclusions

In this study, an ISMS was employed to obtain an automatically stable WHBP without the need for residue filtration. Given colloid milling is a promising technology for plant-based beverage preparation and dietary fiber modification, colloid milling was chosen as a comparison for ISMS in this study. Visual observation and SEM analysis showed that colloid milling failed to effectively homogenize the material, while the ISMS sample surfaces were compact and smooth at higher pressures. The TSI values of WHBP prepared by ISMS were much lower, demonstrating ISMS can improve the stability of WHBP. WHBP produced by colloid milling displayed a three-peak particle size distribution pattern, while a single-peak pattern was evident after ISMS treatment. A higher shear rate decreased the apparent viscosity values of the WHBP samples, suggesting that WHBP was a shear-thinning fluid. According to CLMS, ISMS can successfully improve homogenization by disrupting the structures of oil bodies, proteins, and starches. The WHBP prepared using ISMS exhibited a higher β-glucan level than the sample obtained via colloid milling, and this increased significantly at a higher ISMS treatment pressure. For the HB ratio in WHBP, this study mainly investigated the difference between colloid milling and ISMS under 5%. A higher HB ratio, such as 10%, will be investigated in future research for the purpose of providing a deeper understanding of the difference between colloid milling and ISMS for WHBP. Given that the present study was conducted in the context of a complex food system, a simulation system that consists only of starch and dietary fiber could be built to further study the interaction between specific macromolecules under ISMS in the future. The findings of this study indicate that using ISMS to produce WHBP is viable for enhancing its stability and nutritional value. The ISMS in this paper shows promise for stable, nutritious WHBP production.

## Figures and Tables

**Table 1:** The average particle sizes of the WHBP obtained via colloid milling and ISMS *.

	1 d	3 d	5 d	10 d	20 d	30 d
WHBP_CM-80_	54.26 ± 1.38 ^f^	65.70 ± 0.99 ^e^	67.02 ± 1.65 ^g^	76.50 ± 13.16 ^d^	77.20 ± 8.14 ^f^	220.67 ± 15.95 ^f^
WHBP_CM-300_	35.76 ± 0.27 ^e^	33.72 ± 1.33 ^c^	31.98 ± 0.11 ^c^	31.78 ± 1.75 ^b^	32.84 ± 1.61 ^c^	73.62 ± 4.17 ^e^
WHBP_M-5-30_	34.03 ± 0.40 ^d^	34.43 ± 0.31 ^c^	38.46 ± 0.15 ^e^	34.10 ± 1.17 ^b^	32.20 ± 0.54 ^c^	33.10 ± 0.56 ^b^
WHBP_M-5-60_	29.37 ± 0.47 ^c^	29.60 ± 0.63 ^b^	29.08 ± 0.33 ^b^	29.08 ± 1.00 ^ab^	27.68 ± 0.40 ^b^	29.07 ± 0.40 ^ab^
WHBP_M-5-120_	22.93 ± 0.21 ^a^	24.03 ± 0.06 ^a^	23.43 ± 0.26 ^a^	22.96 ± 0.30 ^a^	22.94 ± 0.54 ^a^	23.40 ± 1.04 ^a^
WHBP_M-10-30_	35.75 ± 0.60 ^e^	47.20 ± 0.96 ^d^	47.87 ± 3.00 ^f^	45.77 ± 2.47 ^c^	52.60 ± 0.81 ^e^	56.60 ± 2.47 ^d^
WHBP_M-10-60_	29.80 ± 0.50 ^c^	34.30 ± 1.48 ^c^	35.37 ± 1.60 ^d^	37.00 ± 1.82 ^b^	37.53 ± 0.58 ^d^	44.08 ± 1.98 ^c^
WHBP_M-10-120_	25.50 ± 0.10 ^b^	29.77 ± 0.12 ^b^	30.00 ± 0.26 ^b^	30.10 ± 0.01 ^ab^	30.20 ± 0.17 ^bc^	28.92 ± 0.47 ^ab^

* Reported results correspond to mean ± standard deviation. Different letters within the same column indicate significant differences (*p* < 0.05).

**Table 2:** Power law model parameter values of the WHBP prepared via colloid milling and ISMS *.

Samples	*k* (Pa·s^n^)	*n*	R^2^
WHBP_CM-80_	0.105 ± 0.001 ^a^	0.682 ± 0.001 ^e^	1.000
WHBP_CM-300_	0.052 ± 0.001 ^a^	0.558 ± 0.001 ^c^	0.999
WHBP_M-5-30_	0.074 ± 0.001 ^a^	0.701 ± 0.001 ^e^	1.000
WHBP_M-5-60_	0.041 ± 0.001 ^a^	0.754 ± 0.001 ^f^	1.000
WHBP_M-5-120_	0.106 ± 0.002 ^a^	0.617 ± 0.005 ^d^	0.999
WHBP_M-10-30_	31.239 ± 0.493 ^d^	0.265 ± 0.005 ^a^	0.996
WHBP_M-10-60_	3.839 ± 0.001 ^c^	0.378 ± 0.001 ^b^	1
WHBP_M-10-120_	1.349 ± 0.174 ^b^	0.682 ± 0.031 ^e^	0.986

* Reported results correspond to mean ± standard deviation. Different letters within the same column indicate significant differences (*p* < 0.05).

## Data Availability

The original contributions presented in the study are included in the article, further inquiries can be directed to the corresponding author.

## References

[1] Wang C., Pan Z., Nima Z., Tang Y., Cai P., Liang J., Deng G., Long H., Yu M. (2011). Starch granule-associated proteins of hull-less barley (*Hordeum vulgare* L.) from the Qinghai-Tibet Plateau in China. J. Sci. Food Agric..

[2] Zhang K., Yang J., Qiao Z., Cao X., Luo Q., Zhao J., Wang F., Zhang W. (2019). Assessment of β-glucans, phenols, flavor and volatile profiles of hulless barley wine originating from highland areas of China. Food Chem..

[3] Wen A., Delaquis P., Stanich K., Toivonen P. (2003). Antilisterial activity of selected phenolic acids. Food Microbiol..

[4] Weng C., Yen G. (2012). Chemopreventive effects of dietary phytochemicals against cancer invasion and metastasis: Phenolic acids, monophenol, polyphenol, and their derivatives. Cancer Treat. Rev..

[5] Moza J., Gujral H.S. (2016). Starch digestibility and bioactivity of high altitude hulless barley. Food Chem..

[6] Guo T., Horvath C., Chen L., Chen J., Zheng B. (2020). Understanding the nutrient composition and nutritional functions of highland barley (Qingke): A review. Trends Food Sci. Technol..

[7] Mert I.D. (2020). The applications of microfluidization in cereals and cereal-based products: An overview. Crit. Rev. Food Sci. Nutr..

[8] Li Y., Deng L., Dai T., Li Y., Chen J., Liu W., Liu C. (2022). Microfluidization: A promising food processing technology and its challenges in industrial application. Food Control.

[9] Kumar A., Dhiman A., Suhag R., Sehrawat R., Upadhyay A., McClements D.J. (2022). Comprehensive review on potential applications of microfluidization in food processing. Food Sci. Biotechnol..

[10] De Bondt Y., Rosa-Sibakov N., Liberloo I., Roye C., Van de Walle D., Dewettinck K., Goos P., Nordlund E., Courtin C.M. (2020). Study into the effect of microfluidisation processing parameters on the physicochemical properties of wheat (*Triticum aestivum* L.) bran. Food Chem..

[11] Li Y., Chen M., Deng L., Liang Y., Liu Y., Liu W., Chen J., Liu C. (2021). Whole soybean milk produced by a novel industry-scale micofluidizer system without soaking and filtering. J. Food Eng..

[12] Dai T., Shuai X., Chen J., Li C., Wang J., Liu W., Liu C., Wang R. (2022). Whole peanut milk prepared by an industry-scale microfluidization system: Physical stability, microstructure, and flavor properties. LWT.

[13] Guo X., McClements D.J., Chen J., He X., Liu W., Dai T., Liu C. (2021). The nutritional and physicochemical properties of whole corn slurry prepared by a novel industry-scale microfluidizer system. LWT.

[14] Lu Y., Kokje T., Schutyser M.A.I., Zhang L. (2022). The effect of colloid milling on the microstructure and functional properties of asparagus dietary fibre concentrates. LWT.

[15] Qi X., Dong Y.n., Wang H., Wang C., Li F. (2017). Application of Turbiscan in the homoaggregation and heteroaggregation of copper nanoparticles. Colloids Surf. A Physicochem. Eng. Asp..

[16] Zuo F., Peng X., Shi X., Guo S. (2016). Effects of high-temperature pressure cooking and traditional cooking on soymilk: Protein particles formation and sensory quality. Food Chem..

[17] Li R., Cao H., Wang Y., Song H., Huang K., Zhang Y., Sun Q., Sun Z., Guan X. (2023). Improving physicochemical stability of highland barley-based milk by the addition of endogenous β-glucan. Food Hydrocoll..

[18] Prins A., Shewry P., Lovegrove A. (2023). Analysis of mixed linkage β-glucan content and structure in different wheat flour milling fractions. J. Cereal Sci..

[19] Gomes M.H.G., Kurozawa L.E. (2023). Performance of rice protein hydrolysates as a stabilizing agent on oil-in-water emulsions. Food Res. Int..

[20] Llinares R., Santos J., Trujillo-Cayado L.A., Ramírez P., Muñoz J. (2018). Enhancing rosemary oil-in-water microfluidized nanoemulsion properties through formulation optimization by response surface methodology. LWT.

[21] Ishii T., Matsumiya K., Nambu Y., Samoto M., Yanagisawa M., Matsumura Y. (2017). Interfacial and emulsifying properties of crude and purified soybean oil bodies. Food Struct..

[22] Peng X., Ren C., Guo S. (2016). Particle formation and gelation of soymilk: Effect of heat. Trends Food Sci. Technol..

[23] Bernat N., Cháfer M., Rodríguez-García J., Chiralt A., González-Martínez C. (2015). Effect of high pressure homogenisation and heat treatment on physical properties and stability of almond and hazelnut milks. LWT-Food Sci. Technol..

[24] Chen B., Cai Y., Liu T., Huang L., Deng X., Zhao Q., Zhao M. (2019). Improvements in physicochemical and emulsifying properties of insoluble soybean fiber by physical-chemical treatments. Food Hydrocoll..

[25] Huang L., Cai Y., Liu T., Zhao X., Chen B., Long Z., Zhao M., Deng X., Zhao Q. (2019). Stability of emulsion stabilized by low-concentration soybean protein isolate: Effects of insoluble soybean fiber. Food Hydrocoll..

[26] Luo X., Wang Q., Fang D., Zhuang W., Chen C., Jiang W., Zheng Y. (2018). Modification of insoluble dietary fibers from bamboo shoot shell: Structural characterization and functional properties. Int. J. Biol. Macromol..

[27] Mercan E., Sert D., Akın N. (2018). Effect of high-pressure homogenisation on viscosity, particle size and microbiological characteristics of skim and whole milk concentrates. Int. Dairy J..

[28] Wang N., Wu L., Huang S., Zhang Y., Zhang F., Zheng J. (2021). Combination treatment of bamboo shoot dietary fiber and dynamic high-pressure microfluidization on rice starch: Influence on physicochemical, structural, and in vitro digestion properties. Food Chem..

[29] Zhou S., Hong Y., Gu Z., Cheng L., Li Z., Li C. (2020). Effect of heat-moisture treatment on the in vitro digestibility and physicochemical properties of starch-hydrocolloid complexes. Food Hydrocoll..

[30] He X., Luo S., Chen M., Xia W., Chen J., Liu C. (2020). Effect of industry-scale microfluidization on structural and physicochemical properties of potato starch. Innov. Food Sci. Emerg. Technol..

[31] Tu Z., Chen L., Wang H., Ruan C., Zhang L., Kou Y. (2014). Effect of fermentation and dynamic high pressure microfluidization on dietary fibre of soybean residue. J. Food Sci. Technol..

[32] Chen J., Gao D., Yang L., Gao Y. (2013). Effect of microfluidization process on the functional properties of insoluble dietary fiber. Food Res. Int..

[33] Liu C., Liang R., Dai T., Ye J., Zeng Z., Luo S., Chen J. (2016). Effect of dynamic high pressure microfluidization modified insoluble dietary fiber on gelatinization and rheology of rice starch. Food Hydrocoll..

